# Disclosure of HIV status to children in resource-limited settings: a systematic review

**DOI:** 10.7448/IAS.16.1.18466

**Published:** 2013-05-27

**Authors:** Rachel C Vreeman, Anna Maria Gramelspacher, Peter O Gisore, Michael L Scanlon, Winstone M Nyandiko

**Affiliations:** 1 Children's Health Services Research, Department of Pediatrics, Indiana University School of Medicine, Indianapolis, IN, USA; 2 USAID-Academic Model Providing Access to Healthcare (AMPATH), Eldoret, Kenya; 3 Department of Child Health and Paediatrics, School of Medicine, Moi University College of Health Sciences, Eldoret, Kenya

**Keywords:** HIV, disclosure, children, resource-limited settings, systematic review

## Abstract

**Introduction:**

Informing children of their own HIV status is an important aspect of long-term disease management, yet there is little evidence of how and when this type of disclosure takes place in resource-limited settings and its impact.

**Methods:**

MEDLINE, EMBASE and Cochrane Databases were searched for the terms hiv AND disclos* AND (child* OR adolesc*). We reviewed 934 article citations and the references of relevant articles to find articles describing disclosure to children and adolescents in resource-limited settings. Data were extracted regarding prevalence of disclosure, factors influencing disclosure, process of disclosure and impact of disclosure on children and caregivers.

**Results:**

Thirty-two articles met the inclusion criteria, with 16 reporting prevalence of disclosure. Of these 16 studies, proportions of disclosed children ranged from 0 to 69.2%. Important factors influencing disclosure included the child's age and perceived ability to understand the meaning of HIV infection and factors related to caregivers, such as education level, openness about their own HIV status and beliefs about children's capacities. Common barriers to disclosure were fear that the child would disclose HIV status to others, fear of stigma and concerns for children's emotional or physical health. Disclosure was mostly led by caregivers and conceptualized as a one-time event, while others described it as a gradual process. Few studies measured the impact of disclosure on children. Findings suggested adherence to antiretroviral therapy (ART) improved post-disclosure but the emotional and psychological effects of disclosure were variable.

**Conclusions:**

Most studies show that a minority of HIV-infected children in resource-limited settings know his/her HIV status. While caregivers identify many factors that influence disclosure, studies suggest both positive and negative effects for children. More research is needed to implement age- and culture-appropriate disclosure in resource-limited settings.

## Introduction

Of the 3.4 million children under the age of 15 years living with HIV worldwide [1], almost 90% live in sub-Saharan Africa [2]. Increasing numbers of these children have access to antiretroviral therapy (ART) and improved prognoses for survival [3]. This requires healthcare systems in resource-limited settings to address challenges, such as maintaining medication adherence, responding to the psychosocial implications of HIV infection, life-skills training and supporting long-term disease management. As more of these children reach adolescence and adulthood, another important challenge is determining how and when to inform children about their HIV status [4,5].

One definition of disclosure refers to a child gaining knowledge of his/her HIV status [6]. For the purposes of this review, we focus on this type of disclosure, while acknowledging paediatric disclosure can also refer to disclosure of caregivers’ HIV status to children [7,8] or a child's disclosure of their own HIV status to others [9,10]. Recommendations for disclosure in the United States endorse a gradual process of giving children age-appropriate information regarding their illness, leading to full disclosure when the child has the cognitive and emotional maturity to process this information [4,6,11,12].

The effects of disclosure are not well studied. While some studies from resource-rich settings show that disclosure is associated with higher self-esteem, fewer symptoms of depression, improved adherence and higher CD4 counts [13–17], other studies do not [18–21]. Furthermore, many studies to date have utilized cross-sectional designs and cannot adequately assess the impact of disclosure on clinical or psychosocial characteristics [6]. Other studies suggest that HIV-infected children who know their status may be better able to seek social support, have improved coping skills [11,22] and practice safer sexual practices to prevent secondary transmission [23,24].

Disclosure is crucial to long-term disease management [12], yet how and when caregivers and healthcare professionals in resource-limited settings disclose to children are not well-characterized and the number of children that know their status is generally thought to be low. Moreover, many of these settings currently lack standardized, culturally appropriate guidelines and resources for undertaking disclosure [25]. While organizations such as the World Health Organization and Médecins Sans Frontières have published recommendations for disclosure of HIV status to children, they do not have a broad evidence base and are not context-specific with considerations of different cultural views on age, maturity and psychosocial development [26–28]. This systematic review aims to estimate the prevalence of HIV disclosure among children in low- and middle-income countries, to examine factors influencing paediatric disclosure, including barriers to and advantages of disclosure, and to assess the impact of disclosure on children's physical and emotional health in these settings.

## Methods

We searched several bibliographic databases, including MEDLINE (January 1, 1966–November 23, 2011), EMBASE (Inception–October 30, 2011), Cochrane Central Register of Controlled Trials (CENTRAL) (Inception–November 23, 2011) and Cochrane Database of Systematic Reviews (Inception–November 23, 2011). We used the search strategy: hiv AND disclos* AND (child* OR adolesc*). Two authors (RCV and AMG) reviewed the titles of all returned articles to determine which studies examined HIV status disclosure to children in resource-limited settings. A structured data extraction tool was used to evaluate all articles. Search terms were only entered in English; articles written in English, French, Spanish, or Portuguese were reviewed. Articles were immediately excluded if they did not involve HIV disclosure, children or adolescents, were not conducted in a resource-limited setting, or were not in English, French, Spanish, or Portuguese. We also searched the bibliographies of the retrieved studies and relevant review articles.

Two authors (RCV and AMG) independently reviewed the articles to determine inclusion. Disagreements were resolved by consensus. For inclusion, the study needed to describe disclosure of HIV status to HIV-infected children or adolescents aged 18 years or less. In addition, the studies needed to be conducted in a resource-limited setting, defined as a low- or middle-income country using the World Bank classification [29]. Studies describing populations of HIV-infected individuals over 18 years of age were included if they also had data on individuals younger than 18 years. Studies that focused only on disclosure of the parent's HIV status to children were excluded, as were studies that focused on disclosure of a child's HIV status to community members, schools, or other individuals, without disclosure to the child. Each article was analyzed to determine the sample characteristics, study setting, definition of and prevalence of disclosure, reasons for and against disclosure, outcomes of disclosure, and the process and method of disclosure.

## Results

### Studies of paediatric HIV disclosure in resource-limited settings

**Figure 1:**
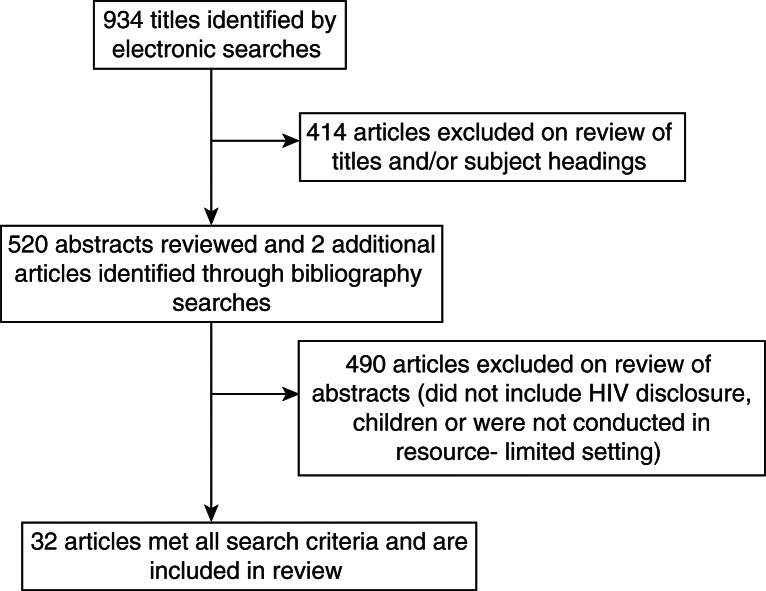
Flow diagram of phases of paediatric disclosure systematic review.

The systematic literature search identified 934 articles (Figure 1). Once articles whose titles alone indicated that they did not address disclosure to HIV-infected children were excluded, 520 articles remained, for which abstracts and then full-text articles were reviewed. Two additional studies were identified through searches of bibliographies [30,31]. Thirty-two articles met all search criteria and were included in this systematic review.

From the thirty-two articles describing HIV disclosure to paediatric patients in resource-limited settings, we extracted data on the location, setting, sample size, study design, population characteristics and prevalence of disclosure (Table 1). Two articles described the same study population [32,33] and therefore were considered a single-study population for this review; however, we make reference to both articles where appropriate due to unique findings. Twenty-one of the studies were conducted in Africa [30,32,34–51] with the remaining primarily from Asia and South America. Most studies relied on small samples, with the largest two studies including 492 HIV-infected children and adolescents [48] and 390 caregivers [44]. Eighteen studies used a qualitative study design including interviews or focus groups [30,32,34,35,37,39–43,47,49,51–56]. Six studies included only children and adolescents [17,31,48,50,53,57] and eight studies included only caregivers (parents or guardians) [25,37,38,42,45,51,55,58]. Ten studies included children and caregivers [30,36,40,41,44,49,52,54,56,59], while seven studies also included healthcare professionals [32,34,35,39,43,46,47].

**Table 1:** Study characteristics

Study	Title	Location	Setting	Study design	Population	Sample size (N)	Children's age range and/or mean or median age (SD)	Proportions of disclosed children
Abadia-Barrero and Larusso, 2006	*The disclosure model versus a developmental illness experience model for children and adolescents living with HIV/AIDS in Sao Paulo, Brazil*	Sao Paulo, Brazil	Home	Qualitative	Children	36	1–15 years	N/A
Arun *et al*., 2009	*Disclosure of the HIV infection status in children*	New Delhi, India	Hospital	Qualitative	Caregivers	50	Mean: 8.98 (0.42)	14%
Bhattacharya *et al*., 2010	*Patterns of diagnosis disclosure and its correlates in HIV-infected North Indian children*	Northern India	Hospital	Cross-sectional	Caregiver-child dyads	290 (145 children, 145 caregivers)	>5 years; mean: 9.1 (2.5)	41.4%
Biadgilign *et al*., 2009	*Barriers and facilitators to antiretroviral medication adherence among HIV-infected paediatric patients in Ethiopia: a qualitative study*	Addis Ababa, Ethiopia	Hospital	Qualitative	Caregivers, health professionals	26 (12 caregivers, 14 health professionals)	1–14 years; mean: 8.52 (2.97)	N/A
Biadgilign *et al*., 2011	*Factors associated with HIV/AIDS diagnostic disclosure to HIV infected children receiving HAART: a multi-center study in Addis Ababa, Ethiopia*	Addis Ababa, Ethiopia	Hospital	Cross-sectional	Caregiver-child dyads	780 (390 children, 390 caregivers)	1–14 years; mean: 8.52 (2.97)	17.4% (by age: 5.9% 0–5 years, 41.2% 6–9 years, 52.9% 10–14 years)
Bikaako-Kajura *et al*., 2006	*Disclosure of HIV status and adherence to daily drugs regimens among HIV-infected children in Uganda*	Kampala, Uganda	Clinic	Qualitative	Caregiver-child dyads	84 (42 children and 42 caregivers)	5–17 years; median: 12	29% complete parental disclosure, 38% partial disclosure
Boon-Yashidi *et al*., 2005	*Diagnosis disclosure in HIV-infected Thai Children*	Bangkok, Thailand	Hospital	Qualitative	Children and caregivers	115 (19 children, 96 caregivers)	5–15 years; mean: 9.6	19.8% (in sample of 96 children. Only disclosed sample – 19 children – were included in qualitative analysis)
Brown *et al*., 2011	*Disclosure of HIV status to infected children in a Nigerian HIV care programme*	Ibadan, Nigeria	Clinic	Cross-sectional	Caregivers	96	6–14 years; mean: 8.8 (2.2)	13.5%
*Corneli *et al*., 2009	*The role of disclosure in relation to assent to participate in HIV-related research among HIV-infected youth: a formative study*	Kinshasa, DRC	Clinic	Qualitative	Children, caregivers, health professionals	72 (19 children, 36 caregivers, 17 health professionals)	11–21 years; median: 16	N/A
De Baets *et al*., 2008	*HIV disclosure and discussions about grief with Shona children: a comparison between healthcare workers and community members in Eastern Zimbabwe*	Eastern Zimbabwe	Hospital and clinic	Qualitative	Health professionals, community members	195 (64 health professionals, 131 community members)	Not reported	N/A
Demmer, 2011	*Experiences of families caring for an HIV-infected child in KwaZulu-Natal, South Africa: an exploratory study*	KwaZulu-Natal, South Africa	Clinic and home	Qualitative	Caregivers, health professionals	25 (13 caregivers, 12 health professionals)	Not reported	N/A
Feinstein *et al*., 2010	*Effect of disclosure on HIV status to children receiving ART on six-month virologic suppression*	Soweto, South Africa	Unspecified	Prospective cohort	Children	492	4–18 years	3% (children aged 4–6 years); 17% (children aged 7–10 years); 77% (children ≥11 years)
Ferris *et al*., 2007	*The influence of disclosure of HIV diagnosis on time to disease progression in a cohort of Romanian children and teens*	Constanta, Romania	Clinic	Retrospective cohort	Children	325	5–17 years; mean: 13.5 (1.5)	69.2%
Fetzer *et al*., 2011	*Barriers to and facilitators of adherence to pediatric antiretroviral therapy in a sub-Saharan setting: insights from a qualitative study*	Kinshasa, DRC	Hospital	Qualitative	Caregiver-child dyads	40 (20 children, 20 caregivers)	9–17 years; median: 14	20.0%
Haberer *et al*., 2011	*Excellent adherence to antiretrovirals in HIV+ Zambian children is compromised by disrupted routine, HIV nondisclosure, and paradoxical income effects*	Lusaka, Zambia	Clinic and home	Prospective cohort	Children	96	Median 6 (IQR 2, 9)	2.0%
Hejoaka, 2009	*Care and secrecy: being a mother of children living with HIV in Burkina Faso*	Burkina Faso	Hospital	Qualitative	Children, caregivers, health professionals	57 (15 children, 20 caregivers, 22 health professionals)	8–18 years	N/A
Kallem *et al*., 2010	*Prevalence and pattern of disclosure of HIV status in HIV-infected children in Ghana*	Accra, Ghana	Hospital	Cross-sectional	Caregiver-child dyads	142 (71 children, 71 caregivers)	8–14 years; mean: 10.42 (1.72)	21%
Kouyoumdjiam *et al*., 2005	*Barriers to disclosure to children with HIV*	Soweto, South Africa	Clinic	Qualitative	Caregivers	17	Not reported	N/A
Lee and Oberdorfer, 2009	*Risk-taking behaviors among vertically HIV-infected adolescents in northern Thailand*	Northern Thailand	Hospital	Qualitative	Children	54	≥ 13 years; median 14.6 (IQR 13.8, 16.1)	N/A
Marques *et al*., 2006	*Disclosure of HIV infection from the perspective of adolescents living with HIV/AIDS and their parents and caregivers*	Sao Paulo and Santos, Brazil	Clinic	Qualitative	Children, caregivers	46 (22 children, 24 caregivers)	10–20 years	N/A
Menon *et al*., 2007	*Mental health and disclosure of HIV status in Zambian adolescents with HIV Infection*	Lusaka, Zambia	Hospital and clinic	Cross-sectional	Children	127	11–15 years; Mean 12.4 (1.4)	37.8%
Moodley *et al*., 2006	*Paediatric HIV disclosure in South Africa – caregivers’ perspectives on discussing HIV with infection children*	Cape Town, South Africa	Hospital	Qualitative	Caregivers	174	0–11 years; median: 3.3	9% overall; 26% in children older than 6 years
Myer *et al*., 2006	*Healthcare providers’ perspectives on discussing HIV status with infected children*	Cape Town, South Africa	Hospital	Qualitative	Health professionals	40	Not reported	N/A
Oberdorfer *et al*., 2006	*Disclosure of HIV/AIDS diagnosis to HIV-infected children in Thailand*	Northern Thailand	Hospital	Cross-sectional	Caregivers	103	6–16 years; mean: 9.5	30%
Petersen *et al*., 2010	*Psychosocial challenges and protective influences for socio-emotional coping of HIV+ adolescents in South Africa: a qualitative investigation*	Durban, South Africa	Hospital	Qualitative	Children, caregivers	40 (25 children, 15 caregivers)	14–16 years	N/A
Punpanich *et al*., 2008	*Understanding the psychosocial needs of HIV-infected children and families: a qualitative study*	Bangkok, Thailand	Hospital	Qualitative	Children, caregivers	69 (34 children, 35 caregivers)	8–16 years; mean: 12.5 (2.2)	N/A
Schaurich, 2011	*Disclosure of AIDS diagnosis to children from the family members’ perspective*	Porto Alegre, Brazil	Clinic	Qualitative	Caregivers	7	Not reported	N/A
*Vaz *et al*., 2008	*The process of HIV status disclosure to HIV-positive youth in Kinshasa, DRC*	Kinshasa, DRC	Clinic	Qualitative	Children, caregivers	40 (19 children, 21 caregivers)	10–21 years; mean: 16.1	N/A
Vaz *et al*., 2010	*Telling children they have HIV: lessons learned from findings of a qualitative study in Sub-Saharan Africa*	Kinshasa, DRC	Clinic	Qualitative	Caregiver-child dyads	16 (7 children, 9 caregivers)	8–17 years	In recruitment, screened 259 children and 8 (3%) had been told their HIV status
Vaz *et al*., 2011	*Patterns of Diagnosis Disclosure of HIV Status to Infected Children in a Sub-Saharan African Setting*	Kinshasa, DRC	Clinic	Qualitative	Caregivers	201	5–17 years; median: 8	N/A
Vreeman *et al*., 2010	*The perceived impact of disclosure of pediatric HIV status on pediatric antiretroviral therapy adherence, child well-being, and social relationships in a resource-limited setting*	Western Kenya	Hospital	Qualitative	Caregivers	120	0–14 years; mean: 6.8	1.7%
Yeap *et al*., 2010	*Factors influencing uptake of HIV care and treatment among children in South Africa – a qualitative study of caregivers and clinic staff*	Gauteng Province, South Africa	Clinic	Qualitative	Caregivers, health professionals	42 (21 caregivers, 21 health professionals)	Not reported	N/A

* Articles describe the same study population.

Some studies defined disclosure generally, using some variant of the concept of children knowing their HIV status [25,34,46,51,56–58], while other studies used a more stringent definition, considering disclosure to have occurred only if there was confirmation that the terms “HIV” or “AIDS” had been used with or specifically mentioned by the child [17,30,32,36,41,45,54,59]. Six studies required a child to have been informed of their status before participating in the study [32,35,40,41,56,57], ensuring that 100% of the sample had been disclosed to, but eliminating the possibility of providing an estimate of disclosure prevalence. Sixteen studies reported proportions of disclosed children for their study population [17,25,30,31,34,36,38,45,48–52,54,58,59], ranging from 0 to 69.2%. Few studies included appropriate sampling methods and scientific design to estimate disclosure prevalence and were not designed to evaluate the impact of disclosure. Most studies reporting disclosure prevalence were cross-sectional in design and employed non-randomized, small, convenience samples of various age ranges of children and adolescents.

### Factors influencing paediatric HIV disclosure

Studies identified multiple factors influencing whether disclosure occurred, many of which were described through qualitative inquiry. Most factors shaping disclosure were at the level of the child or the caregiver (Table 2). Child characteristics considered important to disclosure included the child's age [17,25,31–33,36–42,44,45,51,53,54,58], gender [46], education level [36,39,42,59], medication responsibilities [36], whether the child asks questions [35,37,41,42,51,55,56], and their perceived ability to understand their diagnosis [37,39,40,44,46,47,51,53,54]. The prevalence of disclosure varied most dramatically based on the age of the population, with most children not knowing their HIV status until older ages. For example, in a sample of 492 HIV-infected children in South Africa, 3% of four- to six-year olds knew their status, compared to 17% of seven- to ten-year olds and 77% of those aged 11 years and older [48]. The child's clinical status [32] also influenced disclosure. Receiving ART [31,59], a longer duration of enrolment in clinic or on ART [36,59] and the child having a lower CD4 count [25] were associated with a greater likelihood of disclosure.

**Table 2:** Child and caregiver factors influencing disclosure

Characteristics of the child influencing disclosure	Description of child-related factors
Child's age	More likely to disclose if child is older (Bhattacharya *et al*., 2010; Biadgilign *et al*., 2011; Boon-Yashidi *et al*., 2005; Brown *et al*., 2011; Corneli *et al*., 2009; Demmer, 2011; Ferris *et al*., 2007; Kouyoumdjiam *et al*., 2005; Menon *et al*., 2007; Petersen *et al*., 2010; Schaurich, 2011; Vaz *et al*., 2008; Vaz *et al*., 2010; Vaz *et al*., 2011; Vreeman *et al*., 2010). In multivariate analysis, child's older age was predictor of disclosure (Kallem *et al*., 2010; Oberdorfer *et al*., 2006). Caregivers felt mid-teenage years are appropriate time for disclosure (Arun *et al*., 2009). Caregivers gave median age of 12 years as best age to disclose (Moodley *et al*., 2006). Majority of providers stated 10 years as best age to disclose (Myer *et al*., 2006).
Child's level of maturity/awareness	Varying understanding of illness and therapy over developmental course (Abadia-Barrero and Larusso, 2006). Children <6 years exhibit little understanding of medication and sickness. Unlikely to disclose to them. Children 7–9 years perceive negative connotation with sickness and/or AIDS. Preadolescents have increased awareness of AIDS stigma and negative social values. Adolescents very aware of negative social view of AIDS, but poor understanding of implications of infection. Exhibit cynicism towards HIV-related care (Abadia-Barrero and Larusso, 2006). More likely to disclose if child perceived as being aware of caregiver's illness (Biadgilign *et al*., 2011). Disclosure when children have emotional maturity and intellectual capacity (De Baets *et al*., 2007; Vreeman *et al*., 2010). Disclosure when child able to understand (Demmer, 2011). Advocate disclosure if child able to understand concept of health, disease, and more complex concepts of chronic illness (Myer *et al*., 2006).
Child asks questions about health, disease or HIV	Child's inquisitive or persistent questions makes disclosure more likely (Hejoaka, 2009; Kouyoumdjiam *et al*., 2005; Marques *et al*., 2006; Schaurich, 2011; Vaz *et al*., 2010; Vaz *et al*., 2011; Vreeman *et al*., 2010).
Child's family situation	In multivariate analysis, child having a deceased biological father was a predictor of disclosure (Kallem *et al*., 2010). In multivariate analysis, child not having biological father as main caregiver was a predictor of disclosure (Oberdorfer *et al*., 2006). Caregivers felt disclosure was easier if they were also HIV positive and could show the child that it was possible to have HIV and be healthy (Petersen *et al*., 2010). Sense of concealment within the family: pervasive secrecy may create worry for child and facilitate disclosure (Punpanich *et al*., 2008).
Education/school factors	Enrolment in school increased the likelihood of disclosure. (Bhattacharya *et al*., 2010; Myer *et al*., 2006). In multivariate analysis, higher level of education of the child was a predictor of disclosure. (Kallem *et al*., 2010). Child going to school given as a reason causing caregivers to think more about disclosing (Vaz *et al*., 2011).
Health-related factors	Caregivers report disclosing based on child's health status (Correction pendingneli *et al*., 2009; Vaz *et al*., 2008). Increased likelihood of disclosure was associated with increasing duration since HIV diagnosis and ART initiation and non-perinatal mode of transmission (Bhattacharya *et al*., 2010). In multivariate analysis, self-administration of HIV medication, longer time on ART, and longer time attending clinic were predictors of disclosure (Kallem *et al*., 2010). Disclosure more likely if child on ART (Menon *et al*., 2007). In multivariate analysis, child having most recent CD4 >15% was associated with increased disclosure (Oberdorfer *et al*., 2006).
Feel worried about or unprepared for disclosure	Caregiver beliefs that they are unprepared for questions and process make disclosure difficult (Abadia-Barrero and Larusso, 2006; Boon-Yashidi *et al*., 2005; Demmer, 2011; Kouyoumdjiam *et al*., 2005; Marques *et al*., 2006; Punpanich *et al*., 2008; Schaurich *et al*., 2011; Yeap *et al*., 2010). Caregiver anxiety over disclosure process prevents disclosure (Demmer, 2011). Caregivers believe that they do not know enough about HIV to be able to explain/answer questions prevents disclosure (Kouyoumdjiam *et al*., 2005). Caregivers feel challenged by disclosure emotionally and psychologically; find subject painful and feel not courageous enough to disclose (Kouyoumdjiam *et al*., 2005). Caregivers uncertain how to engage in disclosure process (Punpanich *et al*., 2008).
Fear negative effects of disclosure	Caregiver beliefs that disclosure will cause suffering for the child prevent disclosure (Abadia-Barrero and Larusso, 2006). Caregiver fears of stigma, abandonment, and negative reaction of family and partners prevent disclosure (Demmer, 2011). Caregivers’ fears of shame and stigma prevent disclosure (Bhattacharya *et al*., 2010; Biadgilign *et al*., 2011; Boon-Yashidi *et al*., 2005; Corneli *et al*., 2009; Demmer, 2011; Fetzer *et al*., 2011; Hejoaka, 2009; Kouyoumdjiam et la, 2005; Marques *et al*., 2006; Oberdorfer *et al*., 2006; Punpanich *et al*., 2008; Schaurich, 2011; Vreeman *et al*., 2010). Caregiver fears child will tell others and face discrimination (Abadia-Barrero and Larusso, 2006; Bhattacharya *et al*., 2010; Biadgilign *et al*., 2011; Boon-Yashidi *et al*., 2005; Brown *et al*., 2011; Corneli *et al*., 2009; Fetzer *et al*., 2011; Hejoaka, 2009; Kallem *et al*., 2010; Kouyoumdjiam *et al*., 2005; Moodley *et al*., 2006; Oberdorfer *et al*., 2006; Punpanich *et al*., 2008; Schaurich *et al*., 2011; Vaz *et al*., 2008; Vaz *et al*., 2011; Vreeman *et al*., 2010). Caregivers’ beliefs that knowing status would create emotional stress, sadness or depression for the child prevent disclosure (Abadia-Barrero and Larusso, 2006; Bhattacharya *et al*., 2010; Biadgilign *et al*., 2011; Boon-Yashidi *et al*., 2005; Brown *et al*., 2011; Corneli *et al*., 2009; Demmer 2011; Kallem *et al*., 2010; Marques *et al*., 2006; Moodley *et al*., 2006; Oberdorfer *et al*., 2006; Schaurich, 2011; Vaz *et al*., 2008; Vaz *et al*., 2010; Vaz *et al*., 2011; Vreeman *et al*., 2010; Yeap *et al*., 2010). Caregivers’ beliefs that knowing diagnosis would cause illness to progress more rapidly and/or ART can make people sicker prevent disclosure (Yeap *et al*., 2010).
Belief in keeping HIV concealed/private	Majority of caregivers do not believe in openly discussing HIV and believe status should be concealed (Abadia-Barrero and Larusso, 2006). Caregivers believe in keeping diagnosis secret and do not trust that children can keep diagnosis from others (Fetzer *et al*., 2011).
Other caregiver/family-related factors	Higher education status of caregiver associated with higher likelihood of disclosure (Bhattacharya *et al*., 2010; Biadgilign *et al*., 2011). Caregivers who had discussed their own infection with their child were seven times more likely to have disclosed (Moodley *et al*., 2006). Disclosure was more likely if there were household financial problems (Oberdorfer *et al*., 2006). Disclosure was more likely if caregiver was HIV-infected (Petersen *et al*., 2010).

The characteristics and beliefs of the caregiver further shaped whether and how disclosure took place (Table 2). Caregivers were more likely to disclose if they had a higher level of education [44,46,59] or were more open about their own HIV infection [31,38,40,53]. Caregivers with financial problems [25] and caregivers who were not the father or who were parenting in a context where the father had died [25,36] were also more likely to have disclosed. The caregiver's beliefs about children's ability to understand or about the impact of disclosure further influenced disclosure [32,37,41,43,44,53,56,58]. Variations in disclosure patterns by children's gender were not reported in studies identified in this review.

**Table 3:** Barriers and advantages of disclosure

Barriers to disclosure	Fear child will tell others	Subsequent stigma	Concern for child's emotional or physical health	Believing child unready or too young	Unpreparedness for questions or disclosure process
Studies	Abadia-Barrero and Larusso, 2006*	Bhattacharya *et al*., 2010	Abadia-Barrero and Larusso, 2006*	Abadia-Barrero and Larusso, 2006*	Abadia-Barrero and Larusso, 2006*
	Bhattacharya *et al*., 2010	Biadgilign *et al*., 2011	Bhattacharya *et al*., 2010	Bhattacharya *et al*., 2010	Boon-Yashidi *et al*., 2005*
	Biadgilign *et al*., 2011	Boon-Yashidi *et al*., 2005*	Biadgilign *et al*., 2011	Biadgilign *et al*., 2011	Demmer, 2011*
	Boon-Yashidi *et al*., 2005*	Corneli *et al*., 2009; Vaz *et al*. *2008	Boon-Yashidi *et al*., 2005*	Boon-Yashidi *et al*., 2005*	Kouyoumdjiam *et al*., 2005*
	Brown *et al*., 2011	Demmer, 2011*	Brown *et al*., 2011	Brown *et al*., 2011	Marques *et al*., 2006*
	Corneli *et al*., 2009; Vaz *et al*., 2008*	Fetzer *et al*., 2011*	Corneli *et al*., 2009; Vaz *et al*., *2008	Demmer, 2011*	Punpanich *et al*., 2008*
	Fetzer *et al*., 2011*	Hejoaka, 2009*	Demmer, 2011*	Hejoaka, 2009*	Schaurich, 2011*
	Hejoaka, 2009*	Kouyoumdjiam *et al*., 2005*	Kallem *et al*., 2010	Kallem *et al*., 2010	Yeap *et al*., 2010*
	Kallem *et al*., 2010	Marques *et al*., 2006*	Marques *et al*., 2006*	Kouyoumdjiam *et al*., 2005*	
	Kouyoumdjiam *et al*., 2005*	Oberdorfer *et al*., 2006	Moodley *et al*., 2006*	Myer *et al*., 2006*	
	Moodley *et al*., 2006*	Punpanich *et al*., 2008*	Oberdorfer *et al*., 2006	Oberdorfer *et al*., 2006	
	Oberdorfer *et al*., 2006	*Schaurich, 2011*	Schaurich, 2011*	Punpanich *et al*., 2008*	
	Punpanich *et al*., 2008*	Vreeman *et al*., 2010**	Vaz *et al*., 2010*	Schaurich, 2011*	
	Schaurich, 2011*		Vaz *et al*., 2011*	Vreeman *et al*., 2010*	
	Vaz *et al*., 2011*		Vreeman *et al*., 2010*	Yeap *et al*., 2010*	
	Vreeman *et al*., 2010*		Yeap *et al*., 2010*		
**Advantages of disclosure**	**Improving adherence**	**Improving child's care or treatment**	**Providing answers to child's questions**	**Fulfilling child's right to know**	**Child being able to protect themselves or others**

Studies	Bhattacharya *et al*., 2010	Bhattacharya *et al*., 2010	Bhattacharya *et al*., 2010	Bhattacharya *et al*., 2010	Bhattacharya *et al*., 2010
	Biadgilign *et al*., 2009	Biadgilign *et al*., 2009*	Boon-Yashidi *et al*., 2005*	Corneli *et al*., 2009; Vaz *et al*., *2008	Boon-Yashidi *et al*., 2005*
	Bikaako-Kajura *et* * *al*., 2006	Boon-Yashidi *et al*., 2005*	Brown *et al*., 2011	Moodley *et al*., 2006*	Kallem *et al*., 2010
	Brown *et al*., 2011	Corneli *et al*., 2009; Vaz *et al*., *2008	Hejoaka, 2009*		Marques *et al*., 2006*
	Corneli *et al*., 2009; Vaz *et al*., *2008	Ferris *et al*., 2007	Kallem *et al*., 2010		Vaz *et al*., 2010*
	Fetzer *et al*., 2011*	Hejoaka, 2009*	Kouyoumdjiam *et al*., 2005*		Vaz *et al*., 2011*
	Haberer *et al*., 2011	Kallem *et al*., 2010	Marques *et al*., 2006*		
	Kallem *et al*., 2010	Marques *et al*., 2006*	Punpanich *et al*., 2008*		
	Marques *et al*., 2006*	Moodley *et al*., 2006*	Vaz *et al*., 2010*		
	Oberdorfer *et al*., 2006	Oberdorfer *et al*., 2006			
	Punpanich *et al*., 2008*	Punpanich *et al*., 2008*			
	Vaz *et al*., 2010*	Vaz *et al*., 2010*			
	Vaz *et al*., 2011*	Vaz *et al*., 2011*			
	Vreeman *et al*., 2010*	Vreeman *et al*., 2010*			

* Denotes qualitative study design.

Studies described both barriers preventing the disclosure of HIV status to children in resource-limited settings and potential advantages to disclosure (Table 3). Numerous fears on the part of caregivers and healthcare providers were cited as barriers to disclosure. These included fear the child would disclose to others [25,32,35–38,42,44,45,49,51–55,59], fear of subsequent stigma or negative effects from others knowing the diagnosis [25,32,35,37,44,47,49,51,52,54–56,59], concerns for worsening the child's emotional or physical health [25,32,36,38,41–45,47,51,53–56,59], believing the child is unready or too young [25,35–37,39,43–45,47,51–55,59], feeling unprepared for questions or the disclosure process [43,53–56] and fear of the children's resentment [25,45,53,54,56,59]. In a survey of Nigerian caregivers’ reasons for non-disclosure, caregivers reported fears of the child subsequently telling other children (41%) or family and friends (33.7%), concerns the child was too young to understand (63%), fears the parents would be blamed (26.5%) and fears of psychological disturbance for the child (31%) [45]. Caregivers more often expressed fears of stigma and the child telling others, whereas healthcare providers focused more on children's emotional or physical health and age.

The most common reasons cited as advantages to disclosure were potential improvements in medication adherence [25,30,32,34,36,41,42,45,49–52,56,59] and improving the child's care or treatment [17,25,32,34–36,38,41,42,51,52,54,56,59]. The child's increasing age [32,33,36,39,41,42,54,58,59], being able to answer the child's questions [35–37,41,45,52,54,56,59], fulfilling the child's right to know [32,38,59] and equipping the child to protect others or themselves [36,41,42,54,56,59] were also cited by caregivers as reasons to disclose to children.

### Process of paediatric HIV disclosure in resource-limited settings

While some studies described disclosure as a one-time event, during which a child was told the reason they were taking medicines or was told the name of their diagnosis [25,41,44,53,58,59], other studies described children experiencing partial disclosure before being told they had HIV [30,42,46,55,56]. In several studies, caregivers reported lying to the children about the reason for taking their medicines until after full disclosure [25,42,51,58,59]. This contrasted with the emphasis from disclosed adolescents in Brazil on the importance of children's receiving accurate and complete information about HIV [56]. Vaz *et al*. included a more detailed description of the disclosure process for children in the Democratic Republic of Congo (DRC), where disclosure included giving minimal information about the illness itself, but featured discussion of the medications [33,41]. While caregivers typically conceptualized disclosure as a process, children described disclosure as a discrete event rather than a process, with limited conversations with caregivers and healthcare providers before, during or after disclosure [41]. Parents also described preparatory activities around the day of disclosure, including preparing the child's favourite foods, offering gifts and making sure the child felt loved [33]. In Thailand, caregivers reported that most disclosure events occurred when children were sick and alone with caregivers (89.2%) [25].

Studies endorsed involving both healthcare professionals and the child's parents or caregivers in disclosure. In a majority of studies, caregivers were thought to be the best people to carry out the disclosure process or were reported as the primary discloser [25,30,36–39,41,45,54,58,59]; however, some caregivers wanted healthcare providers to lead disclosure [36,44,57] or preferred that healthcare providers partnered with the child's caregiver [38,39]. In a study from Zimbabwe, 51.3% of caregivers wanted healthcare workers involved with disclosure and 42.3% wanted help from another family member such as a sister or parent [46]. Marques *et al*. reported several instances in which healthcare providers disclosed to children without prior consultation with caregivers who believed their child was not ready or that disclosure by providers was conducted inappropriately [56].

Opinions about the optimal age for disclosure varied. In one study from South Africa, healthcare providers believed six years was appropriate for a general discussion about health issues and ten years was appropriate for HIV-specific disclosure [39]. Caregivers from South Africa endorsed older ages for both events – 11 years for a general discussion and 12 years for HIV-specific information [38]. In Zimbabwe, community members preferred full disclosure at 14–15 years, with partial disclosure at 10–11 years, but healthcare providers preferred younger ages [46]. The caregivers’ preferences generally matched the age at which disclosure was actually done. In Thailand, one study found that being over 10 years was associated with knowing your HIV status [25], and in a small study from the DRC, the median age for disclosure was 15 years, with no children under ten years having been informed of their status; however, only 19 children were included in the study [33]. Two studies reported on caregivers’ and healthcare providers’ desire for protocols, materials or specific guidelines to direct disclosure [37,39].

### Impact of disclosure on children and caregivers

Fourteen studies discussed the impact of disclosure on HIV-infected children; however, no studies evaluated children pre- and post-disclosure [17,25,30–32,35,40,41,43,45,49,50,56,57] (Table 4). The experience of disclosure on children in resource-limited settings was reported through qualitative or descriptive studies, in which disclosure was described as a positive event among the majority of those who went through it [32,56,57]. In Brazil, adolescents characterized disclosure as an essential step in adapting to a “normal” life with HIV and thought disclosure should be done as soon as possible [56]. Among Thai youth aged 13–16 years, 33.3% reported wishing they had been told sooner or much sooner, and 79.6% were satisfied with disclosure process; however, 18.5% wished they had not been told of the diagnosis at all [57].

**Table 4:** Impact of disclosure

Study	Impact of disclosure on children
Bikaako-Kajura *et al*., 2006*	Described improved adherence; disclosure believed to be motivating factor because child understood importance of medication; more positive attitude towards treatment; developed own adherence strategies and/or shared responsibility for treatment.
Brown *et al*., 2011	Caregivers reported improved adherence in 66% of children.
Corneli *et al*., 2009; Vaz 2008*	Improved adherence; knowledge of diagnosis improved adherent behaviours; better able to protect themselves and others; some youths expressed emotional difficulties from disclosure, including sadness, discouragement and fear.
Ferris *et al*., 2007	Significantly more frequent CD4 counts; significantly less likely to experience disease progression and death.
Fetzer *et al*., 2011*	Less frustration with medication-taking; disclosure as a motivating factor for adherent behaviours.
Haberer *et al*., 2011	Significantly fewer missed ART days (compared to undisclosed children).
Hejoaka, 2009*	Improved adherence; children maintained concealment strategies and secrecy.
Lee and Oberdorfer, 2009*	Majority viewed disclosure as a positive event.
Marques *et al*., 2006*	Majority viewed disclosure as a positive event; adolescents felt disclosure had positive long-term psychological impacts and allowed for better self-care and treatment.
Menon *et al*., 2007	Significantly fewer emotional difficulties (compared to undisclosed children).
Oberdorfer *et al*., 2006	Majority of children accepted diagnosis; some reported sadness, anger and rebellion.
Petersen *et al*., 2010*	Negative effects and emotional difficulties included: distress, fear, perceived stigma, internalized stigma, withdrawal from peers, and perceived shortened future. Accepting family social support helped to address these challenges.
Vaz *et al*., 2010*	Negative effects and emotional difficulties included: sadness, worry and perceived stigma; some children reported relief after disclosure and felt disclosure was important.

* Denotes qualitative study design.

The combined evidence from this review suggests that disclosure may play an important role in improving medication adherence and HIV-related outcomes. Four qualitative studies reported adherence improved post-disclosure [30,32,35,49]. In Burkina Faso, youth reported that disclosure enabled them to maintain their HIV treatment, including their ability to conceal the diagnosis from others [35]. As one youth in the DRC described regarding improved adherence: “Having heard explanations made it easy for me to take the medicines. I was told what medicines do in my body. That is why I take them” [49]. Two studies attempted to quantify changes in adherence post-disclosure [45,50]. In a study from Nigeria, among caregivers who had disclosed to children, 64% felt that adherence had improved post-disclosure [45]. A study of paediatric ART adherence in Zambia found that the average number of missed ART days was 38% lower among children who knew their status (*p=*0.001) [50]. Though knowledge of HIV status was strongly associated with age, it remained an independent predictor for adherence when adjusted for age [50]. A study of 325 HIV-infected children in Romania found that disclosed children were less likely to experience disease progression (*p*=0.03), as measured by CD4 count or death [17].

Evidence on the emotional and psychological impact of disclosure was more limited. In the only study identified that quantified the psychological differences between disclosed and non-disclosed children, univariate analysis of 127 children in Zambia found non-disclosed children were more than twice as likely to experience concerning levels of emotional difficulty (OR=2.62, 95% CI: 1.11–6.26) [31]. In qualitative studies, many youth reported initial emotional difficulties, some of which were mitigated over time. In a qualitative study of 25 South African adolescents, almost all found disclosure to be emotionally difficult [40]. In Brazil and the DRC, children reported feeling sadness, grief, and worry upon learning about their HIV infection, but these negative feelings were followed by some feelings of relief [33,41,56]. Congolese children reported feeling calmer after disclosure because knowing their HIV status removed some of the uncertainty surrounding their illness [32]. Despite the negative emotions of sadness and worry, a number of studies reported that children felt that knowing HIV status was important and necessary [33,41,56].

Completing child disclosure also impacted caregivers differently. Some caregivers reported feeling relieved and happy with their decision to disclose [32,56]. Caregivers appreciated sharing the responsibilities of adherence with the child after disclosure [30], and described having less frustration with adherence [49]. Other caregivers reported negative emotional effects after disclosing, such as feeling unprepared for answering children's continuing questions and fear that social stigmatization might result if the child told others [41,56]. No studies measured actual experiences of stigma, discrimination, or social rejection post-disclosure for caregivers or their children. The systematic review found no studies that reported on the impact of disclosure on adolescent sexual behaviours or risk reduction for secondary transmission.

## Discussion

In resource-limited settings, the prevalence of HIV disclosure to children is generally low, even among adolescents. Significant factors influencing disclosure include the child's age, the child's persistent questioning and caregivers’ perceptions of the child's ability to understand and cope up with HIV. Caregivers identify many barriers to disclosure within these settings, the most prominent being fear of stigma and of negative consequences for children's emotional and social well-being. In the midst of caregivers’ worries about disclosure and low prevalence of disclosure, there is only limited evidence to suggest how disclosure will impact children. Some studies assessing children who know their HIV status describe possible improvements in children's medication adherence and emotional health, but other studies describe negative effects. Because the impact of disclosure on children has rarely been assessed quantitatively, research to evaluate how disclosure impacts children's physical, emotional, mental, and social outcomes would improve implementation of age- and culture-appropriate disclosure.

Much of the work assessing disclosure to HIV-infected children in resource-limited settings is qualitative in nature and provides a useful body of literature describing important cultural concepts shaping when, how, and whether children are informed of their HIV status. Reasons given by caregivers for and against disclosure are similar across resource-limited and resource-rich settings with potential benefits weighted against potential harms of disclosure. While the child's age, developmental maturity, and concerns about medication adherence can drive parents and caregivers towards telling the child about their HIV diagnosis, deep-seated fears of the child telling others about the diagnosis and of potential negative emotional consequences for the child also shape caregivers’ decisions about disclosure. As HIV care programmes attempt to engage in disclosure for their growing populations of children, caregivers’ fears of stigma and negative psychosocial effects need to be addressed with appropriate disclosure protocols and procedures.

Learning about their HIV status is clearly an emotional and pivotal point in a child's life. Children describe reacting with sadness and grief, as well as anxiety and worry about what their diagnosis means for the future. In addition, children share their caregivers’ worries about the negative social repercussions that may result if their HIV status is revealed to others. No studies investigated whether fears of stigma translated into actual discrimination post-disclosure. In qualitative studies, disclosure is often described as a positive event, and at least one quantitative study suggested that, despite the immediate burden of learning one's HIV diagnosis, disclosed children actually have better emotional health outcomes compared to their non-disclosed counterparts. Limited evidence also suggests disclosure is associated with better adherence to ART and HIV-related health outcomes, although the effect of disclosure on adherence could not be evaluated.

The compiled evidence of this review suggests that there may be both positive and negative effects from disclosure. Additional qualitative and quantitative studies are needed to investigate how disclosure impacts children, particularly their adherence to ART, and physical, emotional, and social outcomes. Providing appropriate resources and support to caregivers and children through the disclosure process may mitigate any potential negative effects of disclosure. Furthermore, effective strategies to assess and monitor children's clinical and psychosocial well-being throughout the process will also help ensure that children and caregivers receive necessary services. While a number of web-based resources for disclosure exists [28,60,61], their impact has not been evaluated rigorously and merits attention. Investigating how the existing disclosure protocols and procedures may be cross-culturally adapted also deserves further consideration.

There are several limitations to this systematic review that warrant consideration. We may have failed to identify publications from non-traditional or non-Western literature sites, but we attempted to follow the most inclusive and systematic methodology readily available. Few non-English, French, Spanish or Portuguese studies were identified. Publication bias could be a concern; however, we were encouraged by the recent increase in studies from low- and middle-income countries, with all included studies having been published since 2004. In addition, we opted to include only published studies, as the quality of the existing studies was already fraught with limitations. While few studies measured the prevalence of disclosure or the quantitative impact of disclosure, the current understanding of disclosure in these settings is such that qualitative work is critically needed to understand cultural phenomena. The studies were heterogeneous, with inconsistent definitions or processes for disclosure, and few quantitative data about factors related to disclosure. Thus, we could not conduct a meta-analysis at this time for factors related to disclosure or a pooled estimate for the impact of disclosure. Key factors shaping the disclosure process may still be missing from these compiled data. For example, government policies and guidelines in different countries may have legal implications for disclosure. The studies that we summarized did not specifically include discussions of national laws and policies regulating disclosure in particular settings, which may influence the age for disclosure and who can be involved. Studies were also heterogeneous in the ages of study participants, with some studies including only children, others only adolescents, and many including both children and adolescents. Important differences in how disclosure takes place as well as its impact on clinical, emotional, and social outcomes for children and adolescents may be present; however, this review did not investigate this aspect of disclosure because of the limited body of data. Similarly, differences in disclosure patterns by gender could not be assessed in this review. Finally, we excluded studies reporting only on parents’ disclosure of their own HIV status to children or on youth disclosure of their own status to other people. Although these types of disclosure also merit careful consideration, the motivations and potential impacts may be different.

## Conclusions

This systematic review revealed the paucity of data related to disclosure of HIV status to children in low- and middle-income countries. While specific evidence-based recommendations for how and when disclosure should take place are premature, the findings of this review and from the more robust literature available from the United States allow us to make preliminary recommendations for disclosure to children in resource-limited settings and directions for future research. First, disclosure needs to be addressed thoughtfully and proactively as part of long-term disease management. This includes consideration of cultural views about a child's age, maturity level and emotional health and addressing families’ widespread fears about potential HIV-related stigma and discrimination if their child's status is revealed to others. These concerns suggest that improved psychosocial support services in these settings could aid in the disclosure process. Second, there is a need for structured, evidence-based protocols, materials and guidelines for paediatric HIV disclosure that have been rigorously evaluated and incorporate both preparation and on-going communication among children, caregivers and health providers. Existing materials on disclosure should be made available for adaptation, evaluation and broader implementation. Finally, additional research is needed on effective strategies for disclosure and the clinical, emotional and social impact of disclosure on HIV-infected children in resource-limited settings. Longitudinal studies that follow children through the disclosure process will be better able to assess the impact of disclosure and allow clinicians and other providers to deliver appropriate services and support to children and caregivers.
